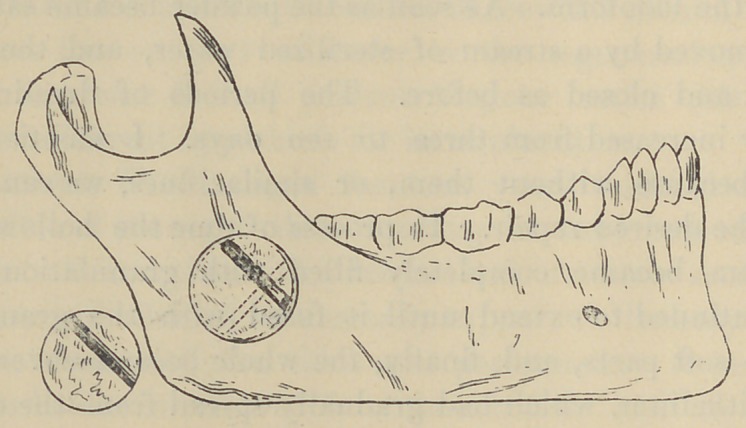# A Contribution to the Study of Bone Repair

**Published:** 1888-09

**Authors:** 


### SELECTIONS.

### Philadelphia County Medical Society.

(Official report. Stated meeting, June 12, 1888. C. B. NANCREDE, M.D., in the chair.)

DR. JOHN S. MILLER read the following paper, entitled

### A CONTRIBUTION TO THE STUDY OF BONE REPAIR.

The recent observations of Macewen (1) have done much to stimulate the study of bone repair, and have not thrown a little light upon the function of the medullary cells in osteogenesis.

The resort to mechanical irritation of the medullary tissues as a means of accelerating bone repair, is an old procedure. Nancrede (2) claims a priority in this for America. As far back as 1793 Eve (3) relates that the lay surgeons of the frontier were wont to make multiple perforation of the external table of the skull where necrosis had followed the Indian mutilation of scalping. And twenty years ago Agnew (4) resorted to the same procedure in a case of injury to the head. A fatal termination of the case, however, by encephalic complication, rendered the experiments incomplete. Reports of success by this procedure have been recently multiplied to an extent which will excuse us from repeating them in detail.

That furthermore, medullary proliferation is not only an element in osteogenesis but is of itself sufficient to that end without periosteal cooperation, is evidenced by the case of Macewen, in which a considerable restoration of the humerus was secured "by bone-transplantation," after a suppurative inflammation had destroyed both the shaft and its periosteum. The date of this observation is 1878.

The patient was a boy, two years of age. A suppurative periostitis of the right humerus of nine weeks' duration had

1. Annals of Surgery, vol. vi. p. 389, et. seq.
2. Internat. Encycl. of Surg., by Ashhurst, vol. 5, p. 8.
3. Remarkable Cases of Surgery, p. 35. Philadelphia, 1857.
4. Loc. cit., p. 301

resulted in total necrosis of the entire diaphysis, and this latter had been removed, leaving a tube of granulation material lining the periosteum. This tube had been kept patent by suitable dressing, until the whole space had become filled with granulation tissue, and had finally become a mass of cicatricial tissue. No bone had grown from this periosteum, except in a small part next the proximal epiphyses, where at the outset the periosteum had been found covered with plaques of adherent osseous tissue. In the remainder there had been no osseous deposit, the result being a flail-like arm, which the patient found so useless that the parents desired its removal.

Macewen determined, however, upon another procedure. An incision was made into the upper third of the arm, exposing the head of the bone, to which was found attached a spike-like process of cartilage. This was removed, leaving as remains of the diaphysis a portion of bone one inch and three-fourths in length. From this point a sulcus about two inches in length was made in a downward direction between the muscles. The former presence of bone was nowhere indicated, and there was no vestige of periosteum, and the sole guide as to the correct position into which the transplant was placed was an anatomical one. Two wedges of bone were then removed from the tibia of a patient aged six years, with anterior curves. The faces of the osseous wedges consisted of the anterior portion of the tibia, along with its periosteum, the wedges gradually tapering toward the posterior of the tibiæ.

After removal they were cut into minute fragments with the chisel, quite irrespective of the periosteum. The bulk of the fragments had no periosteum adhering to them, they having been taken from the interior of the bone.

They were then deposited into the muscular sulcus of the boy's arm, and the tissues drawn over them and carefully adjusted. The wound healed without pus production. Two months after a portion of bone an inch in length and three-quarters of an inch in thickness was found firmly attached to the upper fragment of the humerus.

Two other wedges of bone, larger in size, were similarly dealt with, and inserted two months subsequently to the first graft, and a third couple were placed in position five months after the first. These filled up the gap in the arm to the extent of four and one-quarter inches. The arm then measured six inches in length.

Soon the utility of the arm was greatly restored. Seven years afterward he was seen and examined. The shaft of the humerus was found to have increased in length by one inch and three-quarters, being now seven and three-quarters; and it had increased in circumference to a marked extent, and assumed a somewhat irregular shape. The length of the sound arm had, however, considerbly outstripped the length of the transplanted humerus. He could use the arm for many purposes, taking his food, adjusting his clothes, and many games.

Whether the introduction of proliferating medullary cells into ordinary connective tissue granulations may convert the whole into osseous tissue, or that a few osteoblasts will, so to speak, leaven the whole mass, is a question involving grave doubt, but the affirmative would seem to receive some support from the case which Nancrede ([1]) relates in 1883. An extensive laceration had caused denudation and necrosis of the ulna in two-thirds of its extent. The process of repair had been delayed; he drilled numerous holes through the sequestrum into the medullary canal, and, to quote his own words, "in a few days granulations sprang up from the ulna and fused with the granulations of the soft parts, and, in course of time, the fragment was separated.'

That the procedure in this case had the effect of stimulating osteogenesis from within we can readily believe, but concerning the fusion with granulation tissue without, a more accurate observation than is recorded by Nancrede is desired. Although by analogy we might conceive it possible, inasmuch as repair within the bone is by ossification of an embryonic tissue derived from the connective tissue around the blood-vessels of the medullary spaces. A similar case is reported by Macewen ([2]) in which

1. Transactions of the Philadelphia Academy of Surgery, 1888.
2. Loc. cit., p. 239.

granulations appeared upon a surface of bone completely denuded of its periosteum, and gradually spread until they became united with the granulation tissue at the periphery of the wound. Macewen, however, infers from this observation that

"The periosteum covering a bone may be completely destroyed or permanently removed, yet the denuded bone may not only retain its vitality, but may throw out cells which will cover it and form a new periosteum."

These cases would seem to confirm Macewen's dictum that the periosteum has no part whatever in the regeneration of bone. But the first case I shall present to your notice this evening demands a different hypothesis for its explanation.

The patient, D. M., aged fourteen years, suffered from an osteomyelitis of the right tibia, resulting in total necrosis of its diaphysis. A complete involucrum had formed around the sequestrum and afforded an unsteady support to the body weight. It was covered with the thickened periosteum. A number of fragments had been removed from time to time, and the parents had refused to entertain for him the proposal of amputation. The case, however, when it came into my hands, had become from septic infection so desperate that I was compelled to do something at once.

Exposing the shaft, or rather the involucrum, through its whole length, I made with trephine and saw a fenestrum large enough to permit the removal of the remaining sequestra, and cleared out the whole canal. Both epiphyses were found carious upon their exposed surfaces, and were scraped to the limit of safety. In a few days a superficial necrosis took place upon the inner surface of the tube.

Demarcation was, however, promptly effected by the free use of aluminum acetate (1)—that sheet-anchor in all sloughing wounds—and a layer of fine granulations became the field for any osteogenesis which we might hope to witness. During the long process of repair with the carious epiphysis as a never-failing source of bacterial supply, it was no trifling task to keep this extensive opening dry and sterilized. Furthermore, neither the

---

1. R.—Pot. et alum. sulph., 1 part; plumb. subacet., 5 parts; aquae bull., 100 parts. M. Filtra.

patient, the household, nor the neighborhood could endure frequent dressings without great nervous prostration.

The requirements of the case were successfully met by a mixture of iodoform and starch, in proportions which varied with the changing conditions. The cavity of the wound was filled with this dry powder, and to the whole was applied a closed dressing of gutta percha tissue. The purpose of the starch was to absorb the excess of moisture incident to a closed dressing as well as to dilute the iodoform. As soon as the powder became saturated, it was removed by a stream of sterilized water, and the wound was filled and closed as before. The periods of dressing were gradually increased from three to ten days. I mention these details, because without them, or similar ones, we can wait in vain for the desired repair. In process of time the hollow of the involucrum became completely filled with granulation tissue, which continued to extend until it fused with the granulations from the soft parts, and, finally, the whole became covered with a new epithelium, which had gradually spread from the edges of the wound. The tissues became now denser, and offered more and more support to the body weight until, as you see, he has acquired a very useful limb, and can walk without discomfort.

We must, therefore, infer that a metamorphosis into bone has taken place, and as the original diaphysis was gone with its medullary structure, we can find no osteogenic agent in the result other than the periosteum.

We must draw a similar conclusion from the recent case reported by Ceci:

The patient, a young man, developed an acute osteomyelitis of the left scapula five days after circumcision for inflamed phimosis. One month later, Ceci ([1]) extirpated the bone, making the usual L-flap. The periosteum was left intact as far as possible, and the arm was preserved. The patient recovered rapidly, and there was a subsequent regeneration of the bone.

The only possible explanation of this result is by the hypothesis of periosteal agency or co-operation.

The second case which I present is in confirmation of Macewen's proposition that

1. Centralb. f. Chirrurgie, Dec. 17, 1887.

"A portion of bone which has its continuity severed on all sides, and has all its periosteum removed, is capable of living and growing."

This is in contradiction to our inference in the case of the tibia, and can be reconciled only by the assumption that the discovered laws of osteogenesis are of a lower order, subject to some general law of which we are as yet ignorant.

Mrs. L., aged forty-seven years, had suffered with a neuralgia of the maxillaris inferior, for the relief of which all medical means had been exhausted in vain, and which, therefore, left to my option only the dernier resort of neurectomy. The mode of operating was the usual one. The ramus was trephined near the angle of the jaw, the canal was exposed, and about two inches of nerve trunk were drawn out and exsected. The button was, however, returned after having been sterilized in a 1 to 1000 solution of corrosive sublimate, but it was not returned to its old position. With a view of imposing a barrier to the reproduction of the nerve, it was so rotated around its vertical axis that the groove upon its lower surface stood at right angles to the axis of the canal. Not only did the wound close by first intention, but the button grew solidly in its position. Now, the curious thing in this case is, that before trephining I had carefully removed the periosteum, so that the latter can claim no part in the subsequent bone repair. After seven months there has been no return of the disease.

## Figures and Tables

**Figure f1:**